# *In vivo* Distribution and Clearance of Purified Capsular Polysaccharide from *Burkholderia pseudomallei* in a Murine Model

**DOI:** 10.1371/journal.pntd.0005217

**Published:** 2016-12-12

**Authors:** Teerapat Nualnoi, Adam Kirosingh, Sujata G. Pandit, Peter Thorkildson, Paul J. Brett, Mary N. Burtnick, David P. AuCoin

**Affiliations:** 1 Department of Microbiology and Immunology, University of Nevada School of Medicine, Reno, Nevada, United States of America; 2 Department of Microbiology and Immunology, University of South Alabama, Mobile, Alabama, United States of America; Fondation Raoul Follereau, FRANCE

## Abstract

*Burkholderia pseudomallei* is the causative agent of melioidosis, a severe infection prominent in northern Australia and Southeast Asia. The “gold standard” for melioidosis diagnosis is bacterial isolation, which takes several days to complete. The resulting delay in diagnosis leads to delayed treatments, which could result in death. In an attempt to develop better methods for early diagnosis of melioidosis, *B*. *pseudomallei* capsular polysaccharide (CPS) was identified as an important diagnostic biomarker. A rapid lateral flow immunoassay utilizing CPS-specific monoclonal antibody was developed and tested in endemic regions worldwide. However, the *in vivo* fate and clearance of CPS has never been thoroughly investigated. Here, we injected mice with purified CPS intravenously and determined CPS concentrations in serum, urine, and major organs at various intervals. The results indicate that CPS is predominantly eliminated through urine and no CPS accumulation occurs in the major organs. Immunoblot analysis demonstrated that intact CPS was excreted through urine. To understand how a large molecule like CPS was eliminated without degradation, a 3-dimenational structure of CPS was modeled. The predicted CPS structure has a rod-like shape with a small diameter that could allow it to flow through the glomerulus of the kidney. CPS clearance was determined using exponential decay models and the corrected Akaike Information Criterion. The results show that CPS has a relatively short serum half-life of 2.9 to 4.4 hours. Therefore, the presence of CPS in the serum and/or urine suggests active melioidosis infection and provides a marker to monitor treatment of melioidosis.

## Author Summary

An outer membrane component, capsular polysaccharide (CPS), is a virulence factor expressed by many Gram-negative bacteria including *Burkholderia pseudomallei*, the causative agent of melioidosis. Recently, *B*. *pseudomallei* CPS was identified as a useful diagnostic biomarker, leading to the development of a lateral flow immunoassay (LFI) targeting CPS for *B*. *pseudomallei* detection. In this current work, we studied the *in vivo* fate of CPS using a murine model, to better understand the clinical applications and potential limitations of the LFI. Interestingly, we found that *B*. *pseudomallei* CPS has a unique set of characteristics (as compared to other bacterial capsule antigens) including rapid kidney clearance from serum, no deposition in major internal organs, and ability to be cleared without degradation. Clinically, these findings suggest that CPS may be a potential biomarker for detecting active melioidosis and monitoring melioidosis treatment outcome. Additionally, urine may be used as a non-invasive sample for detecting melioidosis.

## Introduction

*Burkholderia pseudomallei* is a Gram-negative, soil-dwelling bacillus and the etiologic pathogen of melioidosis, a severe infection endemic in tropical areas with the highest incidence in Southeast Asia and northern Australia [1]. In early 2016, it was predicted that approximately 165,000 individuals worldwide would suffer from melioidosis, while 89,000 of them would die from the infection [2]. *B*. *pseudomallei* has also been acknowledged as a potential agent of biological warfare and terrorism because of its ability to cause severe disease via airborne transmission [3,4]. Due to the possibly significant impact on public health and the inherent potential for misuse, the Centers for Disease Control and Prevention (CDC) has classified this organism as a Tier 1 select agent [5]. Currently, there is no licensed vaccine available to prevent the infection. In addition, since *B*. *pseudomallei* is resistant to common antibiotics, the success of melioidosis treatment greatly relies on rapid point-of-care diagnosis [6].

At present, bacterial isolation using Ashdown’s selective medium remains the diagnostic gold standard for melioidosis. This technique is only 60% sensitive along with being time consuming, causing treatment delays and increased mortality risk [7]. Rapid diagnostic methods such as latex agglutination, immunofluorescence assay (IFA), ELISA, and PCR have been developed for *B*. *pseudomallei* detection [8]. In addition to these techniques, a lateral flow immunoassay (LFI) targeting the capsular polysaccharide (CPS) of *B*. *pseudomallei* developed by our group has been shown to be one of the most promising methods for rapid point-of-care detection of melioidosis, especially in resource poor settings [8–10].

The LFI uses a murine monoclonal antibody (mAb) specific to CPS to detect the presence of the bacterium (by detecting CPS) in patient samples. Capsular antigens are outer membrane components expressed by many Gram-negative bacteria, and CPS is known to be one of the most important virulence factors for *B*. *pseudomallei*. Structurally, *B*. *pseudomallei* CPS is an unbranched homopolymer of 1, 3-linked 2-*O*-acetyl-6-deoxy-β-D-*manno-*heptopyranose with an approximate molecular weight of 300 kDa [11,12]. Previous animal model studies have found that a CPS-specific antibody provides protection against lethal challenge with *B*. *pseudomallei*, suggesting that CPS is a candidate target for melioidosis vaccine development [12–14]. In addition, a recent study from our laboratory revealed that CPS antigen circulates in the bloodstream during infection; this led us to develop the CPS-targeting LFI [15]. Currently, clinical performance of the LFI is being assessed in several endemic areas. However, relatively little is known about the ultimate fate of CPS *in vivo*. The main focus of this study was to investigate the *in vivo* distribution and clearance of CPS, information that is essential for improving the clinical use of the LFI.

## Materials and Methods

### Purification of CPS

Culture media was inoculated with *B*. *pseudomallei* RR2683 (O-polysaccharide mutant; select agent-exempt strain, originating in the Brett laboratory) and incubated overnight at 37°C with vigorous shaking [12]. Cell pellets were obtained by centrifugation and extracted using a modified hot aqueous-phenol procedure [11]. Purified CPS was obtained as previously described [12].

### Animals and sample collection

Female, 8-week old CD1 mice (Charles River Laboratories, Inc., Frederick, MA) were injected with 200 μL of dPBS (Mediatech, Inc., Manassas, VA) containing 4, 20 or 100 μg of purified CPS via the tail vein. The CPS doses were chosen according to previous research investigating the clearance of capsule components of *Bacillus anthracis* [16]. At 30 min, 2 hours, 4 hours, 8 hours, 12 hours, 1 day, 2 days, 4 days and 8 days post-injection, mice were euthanized using CO_2_ for sample collection. Urine samples were collected just prior to death. Immediately after euthanasia, blood samples were collected via cardiac puncture and sera were separated. Internal organs including lungs, liver, spleen and kidneys were harvested, weighed and homogenized in 2 mL of dPBS using a PRO250 homogenizer (Pro Scientific, Oxford, CT). Homogenates then were centrifuged and supernatants were collected. All samples were stored at -80°C until quantitative ELISAs were performed.

### Ethics statement

The use of laboratory animals in this study was approved by the University of Nevada, Reno Institutional Animal Care and Use Committee (protocol number 00024). All work with animals at the University of Nevada, Reno is performed in conjunction with the Office of Lab Animal Medicine, which adheres to the National Institutes of Health Office of Laboratory Animal Welfare (OLAW) policies and laws (assurance number A3500-01).

### Quantitative antigen-capture ELISA

An antigen-capture (sandwich) ELISA for CPS quantification was developed using CPS-specific mAb 4C4. Isolation of mAb 4C4 was described previously [17]. Microtiter plates were coated overnight with 100 μL of mAb 4C4 (2.5 μg/mL in PBS) at room temperature. The plates were washed with PBS-Tween (PBS containing 0.05% Tween 20) and blocked with a blocking solution (PBS containing 5% skim milk and 0.5% Tween 20) at 37°C for 1 hour. After blocking, the plates were washed with blocking solution, and then incubated at room temperature for 90 min with 100 μL of a twofold serial dilution of samples (sera, urine or supernatants from tissue homogenates) diluted in blocking solution. A standard CPS sample was prepared by spiking purified CPS into untreated samples diluted in blocking solution. The final concentration of the standard CPS samples was 30 ng/mL. The CPS standard then was added to the plates and serially diluted along with samples to generate the standard curve. After incubation, the plates were washed again with blocking solution, incubated with a mAb 4C4-horseradish peroxidase (HRP) conjugate (0.5 μg/mL in blocking solution) at room temperature for 1 hour, followed by washing with PBS-Tween. The plates were developed by adding 100 μL of tetramethylbenzidine (TMB) substrate (KPL, Gaithersburg, MD) into each well. The reaction was stopped with 1 M H_3_PO_4_, and then the optical density was read at 450 nm (OD_450_). CPS concentrations in samples were determined by comparison with the standard curve using an OD_450_ of 0.5 as the endpoint. The limit of detection of the assay is approximately 0.25 ng/mL.

The amounts of CPS in organ homogenates are reported as micrograms CPS per organ. The CPS amounts reported were corrected by subtraction of the amount of CPS found in the plasma volume in each organ [18], and resulting negative values after subtraction were adjusted to zero. The organ analysis results are presented in comparison with CPS amounts present in serum, which were calculated based on the following information: 1) the blood volume of a mouse is estimated at 5.77 mL/100g, 2) half of the blood volume is plasma [19], and 3) the average weight of mice used in the study is 25.9 g.

### Data analysis

The kinetics of CPS clearance from serum was analyzed using four different exponential decay models: 1) a two-parameter monophasic exponential decay model,

$$y=ae^{-bx},$$

where *a* is the *Y* intercept (concentration of CPS present at time zero) and *b* is the rate constant of clearance, 2) a three-parameter monophasic exponential decay model,

$$y=ae^{-bx}+y_{0},$$

where *a* is the *Y* intercept (concentration of CPS present at time zero), *b* is the rate constant of clearance, and *y*_*0*_ is plateau (concentration of CPS persistent in serum), 3) a four-parameter biphasic exponential decay model,

$$y=ae^{-bx}+ce^{-dx},$$

where *a* is the proportion of CPS that clears rapidly during the initial clearance step, *b* is the rate constant of the initial clearance, *c* is the proportion of CPS that clears more slowly, and *d* is the rate constant of slower clearance step, and 4) a five-parameter biphasic exponential decay model,

$$y=ae^{-bx}+ce^{-dx}+y_{0},$$

where *a* is the proportion of CPS that clears rapidly during the initial clearance step, *b* is the rate constant of the initial clearance, *c* is the proportion of CPS that clears more slowly, *d* is the rate constant of slower clearance step, and *y*_*0*_ is plateau (concentration of CPS persistent in serum). The model fitting was carried out using SigmaPlot 13.0 (Systat Software Inc., San Jose, CA). Corrected Akaike's Information Criterion (AICc), a standard for model selection, was used to evaluate how well each model represents the data. The model that best describes the data (lowest AICc value) among the four equations was selected and used to determine the half-life of CPS in serum.

### Western blotting

To analyze excreted CPS, urine samples from mice injected with 100 μg CPS were analyzed by immunoblot analysis. Only samples collected at 30 min, 2 hours, and 8 hours contained enough CPS for the analysis. The samples were diluted in SDS-PAGE sample buffer, treated with proteinase K (Fisher Scientific, Waltham, MA) at 60°C for 1 hour, and boiled for 10 min prior to electrophoresis on 7.5% TGX precast gels (Bio-Rad, Hercules, CA). The volume of each sample loaded onto the gel was adjusted so that an equal amount of CPS (approximately 1 μg) was present in each lane. Western blotting was performed with mini-nitrocellulose transfer packs and a Trans-Blot Turbo transfer system (Bio-Rad). The membranes were blocked with 5% skim milk in TBS-Tween (TBS-T: 50 mM Tris, 150 mM NaCl, 0.1% Tween 20, pH 7.6) at 4°C overnight, followed by incubation with 1 μg/mL of mAb 4C4 for 90 min at room temperature. After washing with TBS-T, the membranes were incubated with an anti-mouse IgG-HRP conjugate (Southern Biotech, Birmingham, AL) for 60 min at room temperature to facilitate detection. The final development was carried out using Pierce ECL Western Blotting Substrate (Pierce Biotechnology, Rockford, IL) and a ChemiDoc XRS imaging system (Bio-Rad).

### Computational model of 3-dimensional structure of CPS

The chemical structure of the *B*. *pseudomallei* CPS antigen was obtained from previously published work [11]. The structure was drawn in ChemDraw Prime version 15.0 (PerkinElmer, Waltham, MA) and exported to ChemBio3D Ultra software (PerkinElmer). Due to limitations of the software in processing a large molecule like CPS, only a fragment of CPS that consists of 100 units of 2-*O*-acetyl-6-deoxy-β-D-*manno-*heptopyranose was built. Three-dimensional (3D) structure of the CPS was predicted using MM2 energy minimization mode implemented in the ChemBio3D software and exported in protein data bank (PDB) format. The PyMOL program (Schrödinger, LLC, www.pymol.org) was used to visualize and analyze the 3D structure of CPS.

### CPS detection by LFI

Active Melioidosis Detect^™^ (AMD^™^) LFI (InBios International, Inc., Seattle, WA) was used to detect excreted CPS in a urine sample. The urine sample was collected from a mouse injected with 4 μg CPS at 30 min post injection. The sandwich ELISA was used to determine the CPS concentration in this sample. The sample then was serially diluted with mouse control urine to yield the desired concentrations of CPS (0.04–625 ng/mL). Each dilution of urine (5 μL) was mixed with 20 μL of chase buffer (included in AMD^™^ kit). The mixture then was applied to the sample pad, followed by an additional 100 μL of chase buffer. The tests were allowed to develop for 15 min. The results were assessed by four examiners in a semi-blinded, randomized manner and photographed. Intensities of the test lines were also quantified using an ESE-Quant lateral flow reader (QIAGEN, Helden, Germany).

## Results

### *In vivo* clearance of CPS

**Fig 1:**
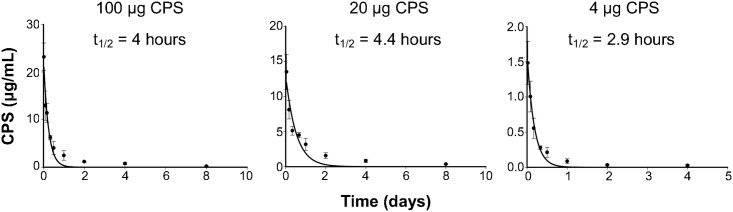
Kinetics for clearance of CPS from serum. Mice were intravenously injected with 100 μg, 20 μg, or 4 μg of CPS. Blood samples were collected at the designated time points, and CPS concentrations in serum samples were determined using quantitative sandwich ELISA. The data were best described by a two-parameter monophasic exponential decay model (*y = ae*^*-bx*^*)*, where *a* is the *Y* intercept and *b* is the rate constant for clearance. Data shown are mean ± standard deviation for five mice per dose per time point. Half-life (t_1/2_) values calculated from the elimination rate constant (*b*) derived from the model fitting were similar for all three doses of CPS. The results demonstrate that CPS is eliminated rapidly from serum with a half-life of 4 hours, 4.4 hours, or 2.9 hours for the doses of 100 μg, 20 μg, or 4 μg CPS, respectively.

Mice were intravenously injected with various doses of purified CPS. At the designated time points, serum and urine samples were collected, and CPS concentrations were determined using antigen capture ELISA. Preliminary analysis was performed to ensure that CPS detection was not significantly affected by the presence of serum or urine (S1 Fig). The kinetics data for CPS in serum were fitted to the exponential decay models, and AICc then was used for model selection. According to the AICc results, clearance of CPS from serum is best described by the two-parameter monophasic exponential decay model (*y = ae*^*-bx*^) (S1 Table). Data analysis showed that CPS was cleared rapidly from serum with a half-life (95% confidence interval) of 4 hours (2.5–6.6 hours), 4.4 hours (2.0–9.7 hours), or 2.9 hours (2.3–3.9 hours), for the doses of 100 μg, 20 μg, or 4 μg, respectively, suggesting that the half-life values derived from all three doses were comparable (Fig 1).

**Fig 2:**
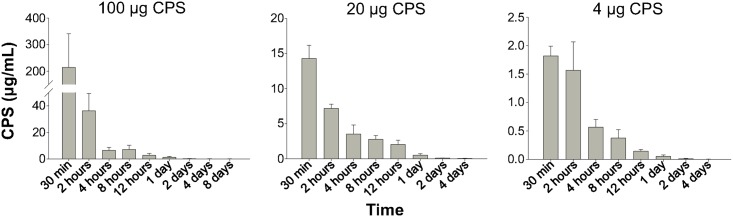
CPS concentrations in urine at various time intervals. At the indicated time points, urine samples were collected from mice intravenously injected with CPS (100, 20 or 4 μg per mouse). CPS concentrations in the samples were determined by quantitative sandwich ELISA. Data shown are mean ± standard deviation for five mice per dose per time point. The results demonstrated that CPS was present in urine shortly after the injection.

Fig 2 shows the concentrations of CPS in urine at different time intervals. The CPS concentrations in urine were found to be highest at 30 min (the first time point of sample collection), and then decreased rapidly, corresponding to the decrease of CPS in serum.

### Organ distribution of CPS

**Fig 3:**
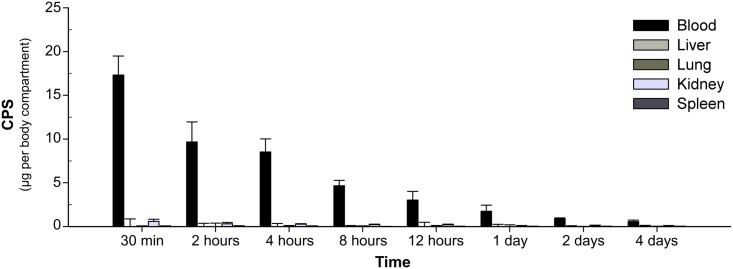
Organ distribution of CPS. Mice were intravenously injected with 100 μg CPS per mouse. Internal organs (lungs, liver, spleen, and kidneys) were collected at various time points post-injection. The organs were homogenized in PBS. CPS amount per organ was determined by quantitative sandwich ELISA. The amount of CPS in blood samples was calculated from the CPS concentration in serum as shown in Fig 1. Data shown are mean ± standard deviation for five mice per time point. The negative values after subtraction of CPS amounts from serum found in each organ were adjusted to zero. The results showed that no significant amount of CPS accumulated in any of the colletced organs.

Kidneys, livers, lungs and spleens were collected from the same CPS-injected mice that were used for the CPS clearance study, but only the samples from mice injected with 100 μg CPS were used. The organs were homogenized in PBS for analysis of CPS concentrations by ELISA. The preliminary analysis showed that the presence of tissue homogenates had no effect on assay performance (S1 Fig). CPS amounts in each organ were reported in comparison with amounts of CPS found in serum (Fig 3). The results showed no significant amounts of CPS deposited in the organs. Since we could not detect CPS in organs from mice injected with 100 μg CPS, the internal organs from mice receiving 20 μg or 4 μg CPS were not analyzed in this study.

### Molecular analysis of excreted CPS

**Fig 4:**
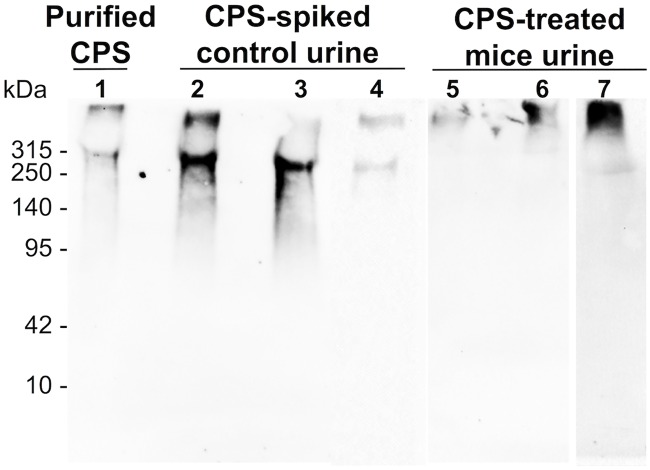
Western blot analysis of excreted CPS. Purified CPS (lane 1) and urine samples (lanes 2–7) were separated on 7.5% SDS-PAGE gels. All samples including purified CPS were incubated with proteinase K at 60°C for 1 hour, followed by boiling for 10 min before loading on the gels. Lanes 2, 3, and 4 were loaded with control urine spiked with CPS and incubated at 37°C for 30 min, 2 hours, and 8 hours, respectively. Lanes 5, 6, and 7 were urine from CPS-injected mice collected at 30 min, 2 hours, and 8 hours post-injection, respectively. The volume of sample loaded into each lane was adjusted to contain an equal amount of CPS, approximately 1 μg/lane. After blotting, membranes were probed with mAb 4C4 (1 μg/mL). Intact CPS was observed in urine samples from CPS-treated mice.

Altogether the results demonstrated that the circulating CPS was eliminated rapidly and predominantly through urine, without accumulation in the major organs. To find out how a large molecule like CPS was excreted, the urine samples from CPS-treated mice were analyzed by Western blot (Fig 4). The Western blots detected full-length CPS in the urine samples, while degraded CPS was not detected. When interpreting these results, however, it is important to note that CPS epitopes might be affected by degradation, so the Western blot analysis might not be able to detect degraded products of CPS. Therefore, it is appropriate to deduce that some (but not necessarily all) of the injected CPS was eliminated without degradation. In some instances, two bands of CPS were observed in our blots. The higher molecular weight band seemed to comprise the largest fraction of CPS in urine samples from these mice; however, how and why this occurred needs to be further investigated (Fig 4).

**Fig 5:**
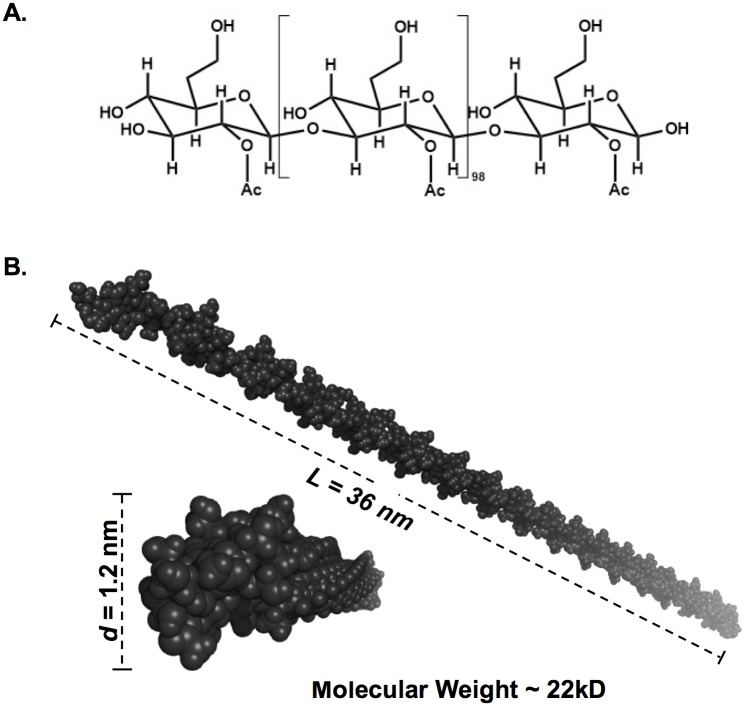
The 3D structure of CPS. The 3D structural model of CPS was predicted from the previously published chemical structure [11] using ChemBio3D software with MM2 energy minimization mode. In this simplified structural assessment, only a ~22 kDa fragment (which consists of 100 units of the *manno*heptose, panel A) was drawn and used for the model prediction. The 3D structural model was visualized using PyMOL (panel B). The predicted structure shows that CPS has a rod-like shape with a dimension (diameter, *d* x length, *L*) of 1.2 nm x 36 nm, which means a full-length CPS molecule may approach 490 nm in length.

Analysis of the total urine protein was used to assess whether or not exogenous CPS affected renal function, which could result in leakage of high molecular weight compounds into urine. The results showed that there was no difference in urine protein profiles between CPS-treated and untreated mice, suggesting that kidney impairment was unlikely the cause of rapid renal excretion of CPS (S1 Fig). In order to explain how CPS was excreted through the kidneys without apparent degradation, a three-dimensional structure of CPS was predicted (Fig 5). However, due to limitations of the software, only a short fragment (~22 kDa) of CPS was constructed (full-length CPS has a molecular weight of ~300 kDa). The computational 3D model demonstrated that CPS has a rod-like shape with a diameter of approximately 1.2 nm. The length of a single molecule of CPS, which was calculated from the length of the 22 kDa fragment model, was approximately 490 nm.

### Detection of excreted CPS by AMD^™^ LFI

**Fig 6:**
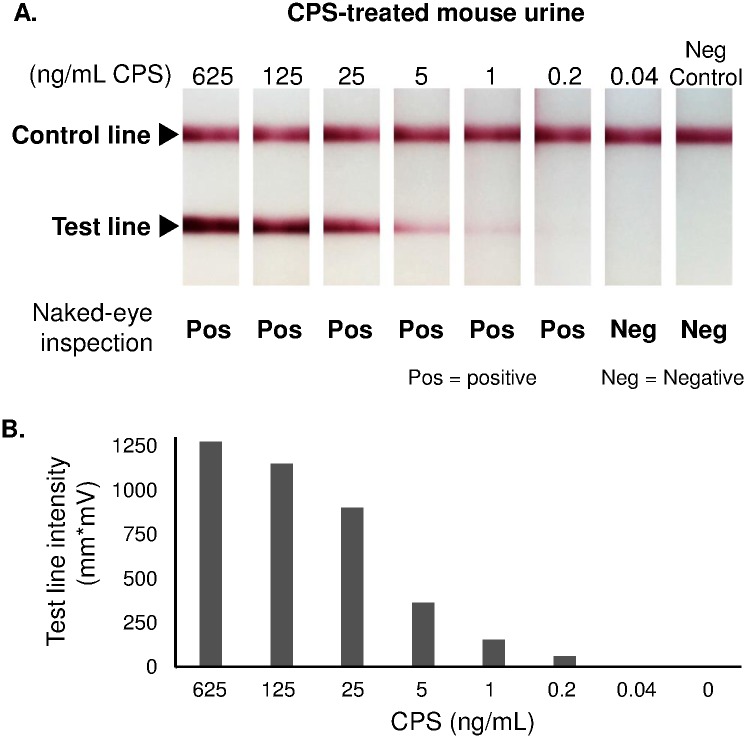
Excreted CPS detection by AMD^™^ LFI. A urine sample from a CPS-treated mouse was serially diluted in mouse control urine to the indicated CPS concentrations. Each concentration of the urine sample then was tested with AMD^™^ LFI. The tests were assessed by four examiners in a randomized, semi-blinded manner (panel A), and by using a lateral flow reader (panel B). The results demonstrated that AMD^™^ LFI could detect excreted CPS as low as 0.2 ng/mL.

Previous experiments demonstrated that CPS is cleared rapidly and predominantly through urine, suggesting that urine has the potential to be used as a non-invasive sample for diagnosis of melioidosis. AMD^™^ LFI is an assay designed to detect *B*. *pseudomallei* by targeting CPS molecules in various types of biological samples. To assess whether or not the sensitivity of AMD^™^ LFI was impacted when it was used to detect excreted CPS in urine samples, serial dilutions of urine from a CPS-treated mouse were tested with the LFI. The results showed that AMD^™^ LFI could detect excreted CPS as low as 0.2 ng/mL (Fig 6), comparable with the AMD^™^ LFI sensitivity reported previously [10].

## Discussion

Like many other pathogenic microorganisms such as *Bacillus anthracis*, *Haemophilus influenzae* type b, *Streptococcus pneumoniae*, and *Cryptococcus neoformans*, *B*. *pseudomallei* expresses a capsular antigen that is shed into the bloodstream during infection [15,20–22]. Based on this finding, immunodiagnostic methods (IFA and LFI, among others) targeting *B*. *pseudomallei* CPS were developed. These diagnostic tools showed similar specificity but higher sensitivity when compared to the ‘gold standard’ diagnostic bacterial culture, suggesting that these techniques have the potential to be used clinically [7,10,23]. To use these tools as a routine diagnostic method, however, it is important to understand the fate of CPS *in vivo*. This could provide insight into the retention and processing time for CPS in a patient during infection, and the potential patient sample types that can be targeted for testing.

To understand how CPS is processed *in vivo*, mice were intravenously injected with purified *B*. *pseudomallei* CPS. CPS concentrations in samples (blood, urine, lungs, liver, spleen, and kidneys) at various time points post-injection were determined using an antigen capture ELISA. The doses of CPS used in this study were chosen according to previous research investigating the clearance of *B*. *anthracis* capsule antigen [16]. We acknowledge that the concentration of CPS in patient serum reported previously was much lower than the lowest dose of CPS we used in this study [10]. However, the range of CPS concentrations reported was from a limited number of serum samples, and was not associated with the stage of infection. In addition, the concentrations reported previously were too low to allow us to collect accurate and sufficient kinetic data in our study. Thus, the experiments were conducted using 4, 20, or 100 μg CPS per mouse.

According to our results, *B*. *pseudomallei* CPS was rapidly cleared from serum with a half-life of 2.9–4.4 h (Fig 1), and not deposited in kidneys, lungs, liver, or spleen (Fig 3). However, relatively high concentrations of CPS were detected in urine shortly after the injection (Fig 2). Notably, at 30 min post-injection, we found that the CPS concentrations in urine were higher than those found in serum for mice receiving 20 or 100 μg CPS (Fig 2). These results indicated that the kidney is the major organ responsible for CPS elimination.

Comparison of our findings with the fate of capsular antigens from other organisms suggests that *B*. *pseudomallei* CPS has a unique set of characteristics. *C*. *neoformans* produces glucuronoxylomannan (GXM) as a major capsule component [24], while *B*. *anthracis* produces a capsule composed of a poly-γ-D-glutamic acid (PGA) polypeptide [25]. *B*. *pseudomallei* capsule is composed of polysaccharides and is somewhat similar in chemical composition to GXM; however, *B*. *pseudomallei* CPS and PGA are apparently similar in geometry, as both of them have a rod-shaped structure [26]. Grinsell et al. demonstrated that GXM has a long serum half-life (~1.6 days) [19]. However, CPS from *B*. *pseudomallei* behaved more like the capsular polypeptide PGA, as both of them showed rapid serum clearance [16]. Previous studies have also found that pneumococcal polysaccharide, GXM and PGA accumulated in many mouse tissues [16,19,27]. In addition, the liver and spleen were found to play important roles in clearance of both GXM and PGA. *B*. *pseudomallei* CPS, however, was not deposited in any mouse organs (kidneys, lungs, spleen and liver), and it was cleared predominantly by the kidneys. Altogether, the results suggest that *B*. *pseudomallei* CPS exhibits certain characteristics distinct from capsular antigens of other previously reported microbes.

Sutherland et al. also showed that PGA was found in urine at high concentrations, which we also found to be true with *B*. *pseudomallei* CPS. The study revealed that PGA was excreted in a degraded form [16]. Since *B*. *pseudomallei* CPS has a high molecular weight, which is much greater than the molecular cutoff for glomerular filtration [28], we anticipated that we would find degraded CPS in urine, as previously seen in the PGA study. However, our results illustrated that a portion (if not all) of circulating *B*. *pseudomallei* CPS was apparently excreted without degradation (Fig 4). The CPS molecule, thus, was further investigated using 3D computer modeling that allowed us calculate a structure of the molecule. The result showed that CPS has a rod-like shape with the dimensions (diameter x length) of 1.2 nm x 490 nm (Fig 5). We found that the structure of CPS resembles a high molecular weight molecule (~350–500 kDa) of a single-walled carbon nanotube (SWCN, dimension = ~1 nm x ~500 nm) [29]. Ruggiero et al. demonstrated that *in vivo* SWCN was excreted rapidly, and predominantly by glomerular filtration of the kidneys, even though its molecular weight exceeds the known glomerular cutoff [29]. As explained by Ruggiero et al., SWCN has a diameter smaller than a glomerular pore (~10 nm), and capillary flow orients the major axis of the rod to align with the glomerular orifice, thereby allowing it to flow through the kidneys. Since *B*. *pseudomallei* CPS molecules and SWCN are geometrically similar, it is possible that CPS and SWCN are eliminated via the kidneys by the same mechanism.

Our results show that *B*. *pseudomallei* CPS has a short serum half-life like PGA from *B*. *anthracis*. However, while the half-life of PGA was dose-dependent, we found that the *B*. *pseudomallei* CPS half-life was dose-independent (Fig 1). This reflected the possibility that CPS and PGA might be eliminated by different mechanisms. We know that PGA was eliminated following degradation; the degradation capacity, however, can be saturated when a treatment dose of PGA exceeds a certain concentration. As a consequence, PGA accumulated faster than it could be cleared; thus the half-life of PGA became longer when the doses were higher (dose-dependence) [16]. For *B*. *pseudomallei* CPS, however, half-life was independent from the treatment doses. We interpret these results to indicate that the CPS elimination capacity was not saturated, at least by the highest concentration used in this study. We found this interpretation fit our proposed mechanism of CPS elimination, in describing that *B*. *pseudomallei* CPS is eliminated passively through glomerular filtration, rather than by degradation or carrier proteins.

Finally, our results have several implications for the clinical use of immunodiagnostics detecting *B*. *pseudomallei* CPS. We know from our previous study that during infection CPS is shed into the blood circulation, indicating that serum can be used as a sample for diagnosis of melioidosis [15]. In this study, we have revealed that CPS can also be detected in urine samples at a high concentration. This finding is consistent with other studies where urine samples from human [10] and non-human primates infected with *B*. *pseudomallei* (K. C. Brittingham, A. Leon, P. A. Braschayko, M. S. Anderson, K. A. Knostman and R. E. Barnewall, presented at the ASM Biodefense and Emerging Diseases Research Meeting, Arlington, VA, 8 to 10 February 2016) were analyzed; results showed a large amount of CPS was present in the urine. In this study, we also discovered that CPS has a half-life of approximately 2.9 to 4.4 h in serum. We noted that our experiments were performed in non-infected animals that have no antibody to CPS. Antibody is known to play an important role in clearing various exogenous antigens, including capsular antigen [30]. Thus, it is possible that, in infected animals or patients with CPS-specific antibody, the serum half-life of CPS could be even shorter than the half-life reported in this study. Since CPS is cleared rapidly from serum by the kidneys, the presence of CPS in serum or urine may suggest an active source of *B*. *pseudomallei* antigen, i.e. acute *B*. *pseudomallei* infection. It also suggests that CPS could be a potential biomarker for monitoring efficacy of melioidosis treatment. In addition, we also found that the AMD^™^ LFI can efficiently detect eliminated CPS in a urine sample (Fig 6). These findings together suggest that perhaps urine, a noninvasive sample that contains a high concentration of CPS, as a sample for melioidosis diagnosis is more appropriate than using serum samples.

In summary, the *in vivo* clearance of *B*. *pseudomallei* CPS has a unique set of characteristics, including i) rapid serum clearance, ii) no significant accumulation in internal organs, iii) potentially passive excretion by glomerular filtration, and iv) presence at a high concentration in urine. Rapid serum clearance of CPS suggests that CPS is a significant biomarker for identifying active melioidosis and monitoring treatment progress. In addition, urine, a noninvasive sample, also has a potential to be used as a clinical specimen for melioidosis diagnosis.

## Supporting Information

S1 Table AICc scores. (DOCX)

S1 Fig Antigen capture ELISA for CPS-spiked serum, urine and tissue homogenates as compared to CPS-spiked PBS. Antigen capture ELISAs were performed with PBS, control serum, control urine, and control tissue homogenates spiked with identical amounts of purified CPS to assess whether different biological sample types affect CPS quantification. The results demonstrated that assay performance was not affected by the different types of samples.
(TIF)

S2 Fig Comparison of total urine protein between control and CPS-treated mice. Molecular weight ladder (lane 1), control urine (lane 2), and urine from CPS-injected mouse collected at 8 hours post-injection (lane 3) were separated on 12% SDS-PAGE. Protein bands were visualized by Coomassie blue staining. The results showed no difference between control urine and urine collected from CPS-treated mice.
(TIF)
