# Supplementary material for: In vivo Distribution and Clearance of Purified Capsular Polysaccharide from Burkholderia pseudomallei in a Murine Model
Source: PLoS Negl Trop Dis. 2016 Dec 12;10(12):e0005217. doi: 10.1371/journal.pntd.0005217 (PMC5179125; doi:10.1371/journal.pntd.0005217)
Supplement: S1 Table — (DOCX) [file pntd.0005217.s001.docx]

**TABLE**

**Table S1. AICc scores. ^a^**

| **Models** | **Injection doses** | | |
| --- | --- | --- | --- |
|  | 100 μg | 20 μg | 4 μg |
| One-parameter monophasic exponential decay ^b^:  *y = ae^-bx^* | 19.44 | 15.17 | -32.55 |
| Two-parameter monophasic exponential decay ^c^:  *y = ae^-bx^ + y_0_* | 23.52 | 20.61 | -29.52 |
| Three-parameter biphasic exponential decay ^d^:  *y = ae^-bx^ + ce^-dx^* | 23.99 | 19.38 | -21.63 |
| Four-parameter biphasic exponential decay ^e^:  *y = ae^-bx^ + ce^-dx^ + y_0_* | 42.23 | 73.93 | 33.27 |

^a^ data were best described by the model with the lowest AICc score

^b^ *a* is the *Y* intercept and *b* is the rate constant of clearance

^c^ *a* is the *Y* intercept, *b* is the rate constant of clearance, and *y_0_* is plateau

^d^ *a* is the proportion of CPS that clears rapidly during the initial clearance step, *b* is the rate constant of the initial clearance, *c* is the proportion of CPS that clears slowly, and *d* is the rate constant of slower clearance step

^e^ *a* is the proportion of CPS that clears rapidly during the initial clearance step, *b* is the rate constant of the initial clearance, *c* is the proportion of CPS that clears slowly, *d* is the rate constant of slower clearance step, and *y_0_* is plateau
