# Supplementary figures and images for: In vivo Distribution and Clearance of Purified Capsular Polysaccharide from Burkholderia pseudomallei in a Murine Model
Source: PLoS Negl Trop Dis. 2016 Dec 12;10(12):e0005217. doi: 10.1371/journal.pntd.0005217 (PMC5179125; doi:10.1371/journal.pntd.0005217)

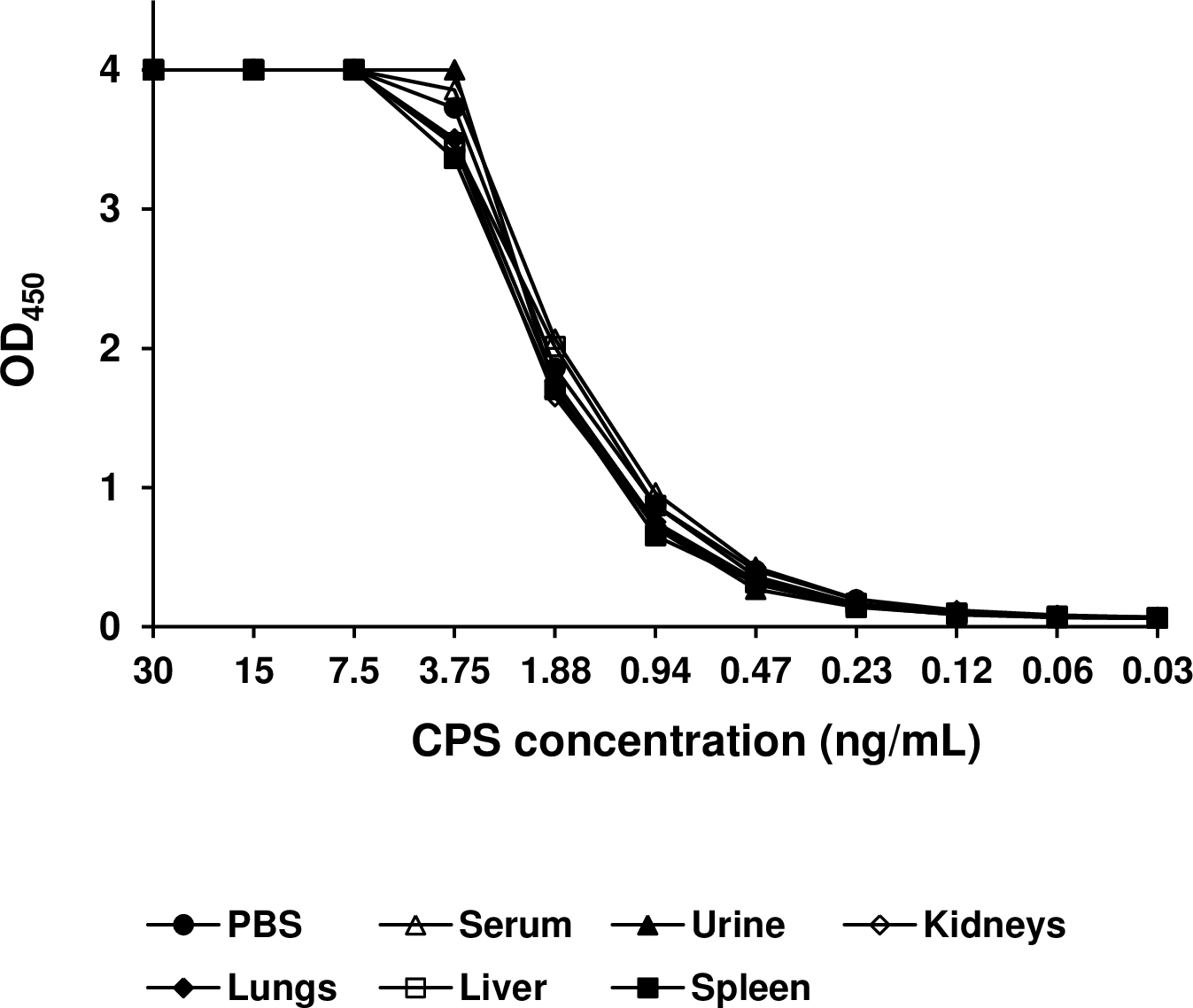

Supplement: S1 Fig — Antigen capture ELISAs were performed with PBS, control serum, control urine, and control tissue homogenates spiked with identical amounts of purified CPS to assess whether different biological sample types affect CPS quantification. The results demonstrated that assay performance was not affected by the different types of samples. (TIF) [file pntd.0005217.s002.tif]

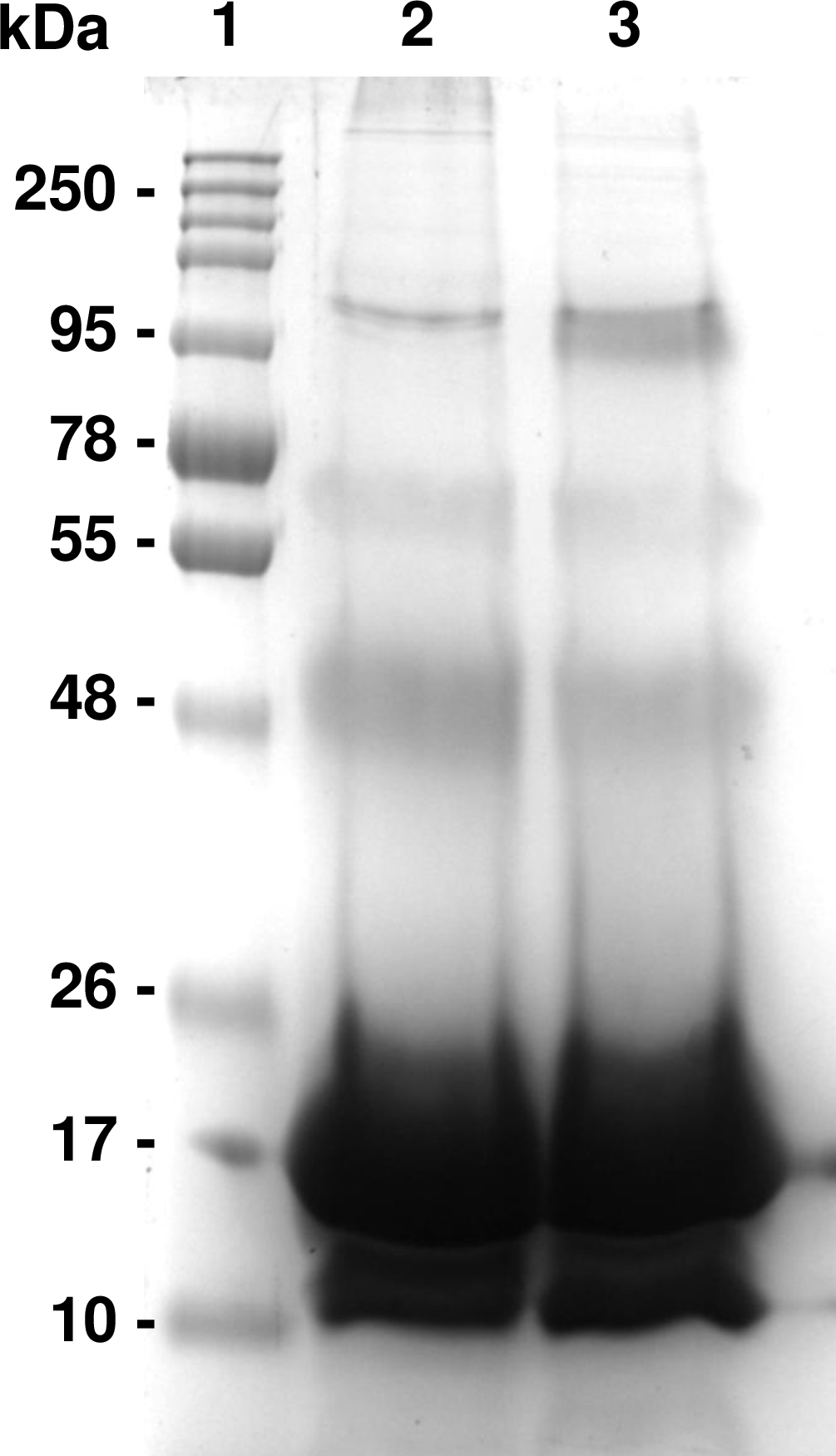

Supplement: S2 Fig — Molecular weight ladder (lane 1), control urine (lane 2), and urine from CPS-injected mouse collected at 8 hours post-injection (lane 3) were separated on 12% SDS-PAGE. Protein bands were visualized by Coomassie blue staining. The results showed no difference between control urine and urine collected from CPS-treated mice. (TIF) [file pntd.0005217.s003.tif]
